# Physico-chemical characterization and partial sequence of a lectin from *Canavalia bonariensis *Lindl seeds

**DOI:** 10.1186/1753-6561-8-S4-P227

**Published:** 2014-10-01

**Authors:** Mayara Silva, Suzete Silva, Kyria Nascimento, Celso Nagano, Benildo Cavada

**Affiliations:** 1 Universidade Federal do Ceará, Fortaleza, CE, Brazil

## 

Lectins are (glycol) proteins that bind specifically and reversibly to carbohydrates. These proteins, in particular those from plant, are important tools in glycobiochemistry and glycobiology. *Canavalia bonariensis *Lindl is a species of Leguminosae family, Papilionoideae subfamily, tribe Phaseoleae, subtribe Diocleinae, native of the southern region of the country. The objective of this work was to purify a lectin from *C. bonariensis *(CaBo) seeds through affinity chromatographic. The process of purification of CaBo (*Canavalia bonariensis *Lectin) was monitored by SDS-PAGE and hemagglutinating activity and showed that the purified lectin is characterized by an electrophoretic profile consists of a higher band with approximately 26 kDa, and two bottom bands with apparent molecular mass of 14 and 12 kDa. The analysis by mass spectrometry indicated that CaBo has a chain with molecular mass of 25,512 kDa and and two subunits (β and γ chains) with molecular mass of 12,999 Da and 12,537 Da, respectively. CaBo also had its primary sequence partially determined by tandem mass spectrometry, obtaining 61% of the total sequence of the protein. CaBo was tested for the thermostability of their hemagglutinating activity after incubation for one hour at different temperatures (40° to 80° C), losing activity only at 80° C after one hour. Regarding its stability at different pH (4.0 to 10.0), CaBo was stable in a pH range between 7.0 and 9.0. The CaBo activity was also affected after serial dilution in the presence of the chelating agent EDTA and it was recovered significantly after addition of CaCl_2 _and MnCl_2 _0,005 mol/L, proving to be dependent of divalent metal cations.

